# Integrated transcriptomic and single-cell RNA-seq analysis identifies CLCNKB, KLK1 and PLEKHA4 as key gene of AKI-to-CKD progression

**DOI:** 10.3389/fimmu.2025.1628962

**Published:** 2025-09-30

**Authors:** Fanhua Zeng, Zhenhua Yang, Zufeng Wang

**Affiliations:** Department of Nephrology, The First Affiliated Hospital of Guangxi Medical University, Nanning, GuangXi, China

**Keywords:** acute kidney injury, chronic kidney disease, biomarkers, drug prediction, single-cell RNA sequencing

## Abstract

**Background:**

Studies have demonstrated a significant connection between acute kidney injury (AKI) and chronic kidney disease (CKD). The purpose of this study was to identify biomarkers linked to the advancement of AKI and CKD, aiming to offer new targets and insights for treating and intervening in these conditions.

**Methods:**

Initially, candidate genes were identified by overlapping the results from differential expression analyses of AKI and CKD. Biomarkers were subsequently identified using machine learning algorithms, receiver operating characteristic curve analysis, expression analysis and experimental verification. Functional enrichment, drug prediction analyses and immune cells infiltration were conducted to investigate the functional mechanisms of the identified biomarkers. Furthermore, single-cell analyses were performed to examine the trends of biomarker expression across different cell types.

**Results:**

CLCNKB, KLK1 and PLEKHA4 were identified as biomarkers by the screening. Subsequently, enrichment analysis showed that CLCNKB was notably enriched in oxidative phosphorylation and the degradation of valine, leucine, and isoleucine in both AKI and CKD datasets. CLCNKB, KLK1 and PLEKHA4 were found to be significantly associated with multiple immune cell types. The regulatory network indicated that PLEKHA4 might play a more important role in the progression of AKI and CKD. Furthermore, it was discovered that CLCNKB, KLK1, and PLEKHA4 are commonly targeted by tetrachlorodibenzodioxin. Finally, in the single-cell data analysis, Type A intercalated cell and Collecting duct-principal cell were identified as the key cells. It was observed that the expression trends of these biomarkers were different under different differentiation states of the key cell subpopulations.

**Conclusion:**

CLCNKB, KLK1 and PLEKHA4 were identified as biomarkers related to the development of AKI and CKD in this study, and new ideas were provided for the research on the potential mechanisms of the progression of AKI and CKD.

## 1 Background

Acute kidney injury (AKI) is identified by a quick loss of kidney function happening within a 48-hour period (1). In medical environments, AKI is typically marked by a rapid increase in serum creatinine and a significant decrease in urine output, often resulting from renal tubular cell necrosis and tissue damage following acute insults such as renal ischemia-reperfusion, exposure to nephrotoxic medications, and sepsis, among other causes (2). Currently, AKI affects 10-15% of all patients in hospitals and up to 50% of those in ICUs, with its prevalence growing annually (3). Furthermore, AKI contributes to long-term chronic kidney damage and accelerates the onset of various complications, including hypertension and cardiovascular disease (4). Alternatively, chronic kidney disease (CKD) is a progressive and lasting disorder identified by the degeneration of renal units, tubular atrophy, interstitial fibrosis, glomerulosclerosis, vascular thinning, and arteriosclerosis (5). A glomerular filtration rate under 60 mL/min/1.73m² for a period exceeding three months defines CKD (6). In addition, the incidence rate of CKD globally is roughly 9.1% (7). Due to its high incidence, significant economic impact, and strong association with morbidity and mortality, CKD represents a major public health concern (8). The clinical management of CKD is hindered by several limitations (9). Therefore, novel insights into the mechanism of AKI and CKD are urgently required to enhance CKD treatment strategies.

Clinically, AKI and CKD are closely interrelated. Atrophy of the tubules and fibrosis in the interstitial area are pathological changes that arise due to inadequate repair mechanisms following AKI, ultimately leading to the development of CKD (10). In China, there are at least 3 million cases of AKI annually, with approximately 50% of survivors subsequently developing CKD (11). Furthermore, individuals with CKD have a higher chance of developing AKI due to pre-existing renal lesions (12). The transition from AKI to CKD is thought to be significantly influenced by the immune-inflammatory response and kidney fibrosis, both of which contribute to persistent renal damage (13). Despite this, the transition from AKI to CKD remains largely unexplored, with the key genes and pathways involved in this intermediary process not yet clearly identified. Hence, it is essential to pinpoint biomarkers related to the progression from AKI to CKD to uncover possible therapeutic targets.

This study utilized transcriptomic and single-cell datasets from public repositories related to AKI and CKD to evaluate biomarkers associated with the progression of these conditions. The assessment was conducted through differential expression analysis, machine learning algorithms, Receiver Operating Characteristic (ROC) curve evaluation, and expression validation. Subsequently, the potential mechanisms of action of these biomarkers in AKI and CKD were explored using biomarker enrichment analysis, immune infiltration analysis, molecular regulatory network construction, and drug prediction. The single-cell data enabled the examination of intercellular communication, leading to the identification of key cellular entities and additional experiments were undertaken to verify the results. Furthermore, we investigated the expression trends of biomarkers in specific cell subsets to elucidate the molecular roles of these biomarkers and their mechanisms in the progression of AKI and CKD. This research aims to offer new perspectives for the early detection and personalized treatment of AKI and CKD patients, thereby reducing the societal burden of kidney diseases.

## 2 Materials and methods

### 2.1 Data source

Datasets related to both AKI and CKD were sourced from the Gene Expression Omnibus (GEO) database (http://www.ncbi.nlm.nih.gov/geo/). GSE139061 (GPL20301) consisted of 39 renal tissue samples from AKI patients and 9 normal renal tissue samples, while GSE30718 (GPL570) included samples included 28 from AKI patients and 11 from healthy kidney tissues, functioning respectively as the training set and validation set of AKI. Similarly, GSE66494 (GPL6480) consisted of 53 renal tissue samples from CKD patients and 8 normal renal tissue samples, while GSE104948 (GPL22945) included 50 CKD patients’ renal tissue samples and 18 normal renal tissue samples, serving respectively as the training set and validation set of CKD. Furthermore, GSE183277 (GPL24676) comprised single-cell RNA sequencing (scRNA-seq) data from kidney cortex tissue samples of 5 AKI patients, 2 CKD patients and 11 normal individuals.

### 2.2 Differential expression analysis

Differentially expressed genes1 (DEGs1) between AKI and normal samples in the GSE139061 dataset were pinpointed by employing the DEseq2 (v 1.38.0) package (14).The dataset was normalized using the estimateSizeFactors function, and genes with counts ≤ 1 were filtered out. DEGs1 were selected with the thresholds of |log_2_fold-change (FC)| > 1.5 and P < 0.05, and the false discovery rate (FDR) was applied to control for multiple comparisons. For the GSE66494 dataset, differential expression analysis between CKD and control samples was performed using the limma package (v3.44.3) (15). Genes with missing values were removed using the na.omit() function. DEGs2 were identified with the same thresholds of log_2_FC > 1.5 and P < 0.05, and FDR correction was applied. Subsequently, DEGs1 and DEGs2 were visualized as volcano plots and heatmaps, displaying only the top 10 in descending order of log2FC for both up- and down-regulated genes. The visualizations were generated through the ggplot2 (v 3.3.2) package (16) and the pheatmap (v 0.7.7) package (17), respectively.

### 2.3 Identification and functional analysis of candidate genes

To identify candidate genes in AKI and CKD progression, the up-regulated genes from DEGs1 and DEGs2, as well as the down-regulated genes from DEGs1 and DEGs2 were separately overlapped using (v 1.7.3) ggvenn package (18). Subsequently, the clusterProfiler package (version 3.16.0) was used to perform Gene Ontology (GO) and Kyoto Encyclopedia of the Genome (KEGG) enrichment analyses on the candidate genes (19). The Benjamini-Hochberg (BH) method was applied to control the FDR, with a significance threshold of pvalueCutoff = 0.05. The top 10 most significantly enriched terms (ranked in ascending order of p-value) from the GO and KEGG analyses were visualized using the enrichplot package (v 1.14.2) (20). To explore protein interactions among the candidate genes, the protein-protein interaction (PPI) network (interaction score > 0.15) was constructed using the Searching for Interacting Genes (STRING, https://www.string-db.org) database and the results were visualized using Cytoscape (v 3.10.2) software (21).

### 2.4 Biomarkers identification and expression analysis

The glmnet (v 4.1.4) package was used to apply the least absolute shrinkage and selection operator (LASSO) method to the candidate genes in the GSE139061 and GSE66494 datasets (22). The parameter family was set as binomial, and 10-fold cross-validation (nfolds = 10) was performed to determine the optimal lambda (λ) value. Potential feature genes were screened based on the lambda.min value for each dataset. Moreover, feature genes were obtained by overlapping the potential feature genes obtained from the GSE139061 and GSE66494 datasets, respectively. Immediately, to evaluate the potential of the feature genes to distinguish AKI samples from control samples, and CKD samples from control samples, these feature genes were subjected to ROC curve analysis using pROC (v 1.18.0) package (23) in the AKI training set and the AKI validation set, the CKD training set and the CKD validation set, respectively, and feature genes with area under the curve (AUC)>0.7 in all four datasets were named as candidate biomarkers. Simultaneously, the candidate biomarkers were subjected to gene expression analysis in the AKI training set and AKI validation set, the CKD training set and the CKD validation set, respectively, and the candidate biomarkers showing a notable difference (P<0.05) between the disease samples and the control samples in the four datasets and a consistent expression trend were selected as the biomarkers for the subsequent analyses.

### 2.5 Gene set enrichment analysis

To investigate the biological roles of biomarkers involved in AKI and CKD, GSEA was performed in the GSE139061 and GSE66494 datasets, respectively. For the analysis, the c2.cp.kegg.v2023.1.Hs.symbols.gmt gene set was acquired from the Molecular Signatures Database (MSigDB, https://www.gsea-msigdb.org/gsea/msigdb/) to act as the background set. First, Spearman correlations between the biomarkers and other genes were calculated using the psych (v 2.2.5) package (24) in the GSE139061 dataset. Subsequently, GSEA for each biomarker was constructed using clusterProfiler (v 3.16.0) package, with significance determined at P <0.05 and |normalized enrichment score (NES)| > 1. The top five pathways in descending order of P-value were visualized using the enrichplot package (v 1.14.2). Similarly, GSEA of the biomarkers was carried out using the same methods and thresholds in the GSE66494 dataset.

### 2.6 Analysis of immune infiltration and cytokines expression

To assess the infiltration of 64 immune cells in disease samples and control samples in the GSE139061 and GSE66494 datasets, respectively. In the case of the GSE139061 dataset, relative abundance was calculated using the xCell (v 1.1.0) package (25), and the proportionate distribution of the 64 immune cells of the AKI samples versus the control samples was visualized using the ggplot2 (v 3.3.2) package. Differences in infiltration scores between AKI samples and control samples in the GSE139061 dataset were then assessed using Wilcoxon test to screen for immune cell types with a significant difference in infiltration (P<0.05), which were named differential immune cells. Subsequently, Spearman correlation analysis was performed using corrplot (v 0.92) package (26) to explore the relationship between differential immune cells and the association between diverse immune cells and biomarkers(|cor| > 0.30, P < 0.05), and correlation heatmaps were plotted to show the results. In addition, immune infiltration and correlation analyses were carried out in the GSE66494 dataset with the same methods and thresholds.

### 2.7 Construction of regulatory networks and drug prediction

Biomarkers targeted by miRNAs were forecasted using the TargetScan (http://www.targetscan.org/) and miRDB (http://mirdb.org/) databases. The transcription factors (TFs) that regulate biomarkers were predicted through the ChEA3 (https://maayanlab.cloud/chea3/) database. Then, the lncRNAs targeting the aforementioned miRNAs were predicted by means of the LncBase (http://carolina.imis.athena-innovation.gr/diana_tools/web/index.php?r=lncba) database. The miRNA-mRNA, TF-mRNA and TF-mRNA-miRNA networks were visualized by using the Cytoscape (v 3.10.2) software, and using the ggplot2 package (v 3.3.2), the lncRNA-miRNA-mRNA network was visualized. Additionally, the Comparative Toxicogenomics Database (CTD, http://ctdbase.org/) was employed to predict drugs targeting biomarkers and Cytoscape version 3.10.2 was employed to plot the biomarker-drug network.

### 2.8 scRNA-seq analysis

Firstly, 5 AKI samples and 11 control samples were selected from the GSE183277 dataset as the AKI single-cell dataset. The AKI single-cell dataset was the “Seurat” package (v 4.1.0) was utilized for quality control (QC) to filter out cells with exceeded 20% of mitochondrial genes, cells with nCount_RNA under 200 and surpassed 30,000 genes, and cells with nFeature_RNA > 200 (27). Then, in light of the GSE183277 dataset, data were normalized by the “NormalizeData” function in the “Seurat” package (v 4.1.0), and highly variable genes (HVGs) were selected by the “FindVariableFeatures” function. Next, the “ScaleData” function in the “Seurat” package (v 4.1.0) was applied to scale data before principal components analysis (PCA). Subsequently, the “JackStraw” function within the “Seurat” package (v 5.0.1) was applied to execute PCA on HVGs. The “ElbowPlot” function within the “Seurat” package (v 4.1.0) was thereafter applied to draw a scree plot of the top 30 principal components (PCs), aiming to identify PCs that notably contributed to variation for subsequent analysis (p < 0.05). Afterward, cell cluster analysis was conducted on cells after dimensionality reduction utilizing “FindNeighbors” and “FindClusters” functions (resolution = 0.2, dimension = 30). Finally, the Seurat package’s FindNeighbors and FindClusters functions were employed to categorize all high-quality cells into various cell clusters using the uniform manifold approximation and projection (UMAP) clustering technique.The FindAllMarkers function was used to identify key marker genes for various populations, and the classical marker genes of relevant cells in the CellMarker (http://xteam.xbio.top/CellMarker/) database were used as the reference gene set to annotate each cell cluster (**Supplementary Table 1**). Additionally, 2 CKD samples and 11 control samples were selected from the GSE183277 dataset as the CKD single-cell dataset and analyzed by scRNA-seq in the same way, with marker genes shown in **Supplementary Table 2**.

### 2.9 Cell communication analysis and identification of key cells

Cellular communication networks between cell types of AKI samples and control samples as well as those between cell types of CKD samples and control samples were analyzed respectively using the CellChat (v 1.6.1) package (28) based on the AKI single-cell dataset and the CKD single-cell dataset. And visualization was carried out by using the patchwork (v 1.3.0) package (29). In addition, key cells were screened and obtained based on the expression situation of biomarkers in cell types within the 2 single-cell datasets.

### 2.10 Pseudotime analysis

To explore the expression changes of biomarkers during the process of cell state transformation, key cells were first extracted respectively based on the AKI single-cell dataset and the CKD single-cell dataset for secondary dimensionality reduction and clustering, and the key cells were reclustered and divided into different cell subpopulations. Following this, the Monocle (v 2.30.0) package was used to conduct cell pseudo-time trajectory analysis on both the AKI and CKD single-cell datasets (30).

### 2.11 Mice models

In this study, male C57BL/6J mice, approximately 8 weeks of age, were utilized. The strain was sourced from the University Model Animal Research Center at Guangxi Medical University. Ethical approval for the use of animals in this research was obtained in compliance with the Guidelines for the Management of Laboratory Animals as stipulated by the Ministry of Science and Technology of the People’s Republic of China, as well as the Guidelines for Ethical Review of Laboratory Animals according to the National Standard GB/T35892–2018 of the People’s Republic of China, and the protocols of the Animal Care and Welfare Committee at Guangxi Medical University (No:202506002). The mice were provided with food and water ad libitum, and the housing environment was maintained at a temperature of 25 ± 2°C with a 12-hour light/dark cycle. The experimental design included three groups of mice, with the model being established through renal artery ischemia-reperfusion surgery. For the intervention study, the C57BL/6J mice were divided into three groups (n = 5 or 6 per group): (1) normal control group; (2) AKI group; and (3) CKD group. Ischemic AKI was experimentally induced using a bilateral ischemia-reperfusion injury (BIRI) model. In this model, mice were anesthetized, and bilateral dorsal incisions were performed to access the kidneys. Both kidneys were then clamped to occlude blood flow for a duration of 30 minutes. CKD was simulated through a unilateral ischemia-reperfusion procedure combined with a contralateral total nephrectomy. Following anesthesia, a left dorsal incision was made to clamp the left kidney, obstructing blood flow for 30 minutes. Subsequently, 14 days post-procedure, a right dorsal incision was executed to facilitate the complete removal of the right kidney (31).

### 2.12 Immunohistochemistry

Kidney tissues were paraffin-embedded and sectioned into 4 μm slices. After deparaffinization and rehydration, antigen retrieval was conducted with EDTA buffer at pH 9.0 for 25 minutes. A 15-minute treatment with 10% hydrogen peroxide was used to block endogenous peroxidase activity, and secondary antibodies were blocked with 5% serum for 30 minutes at room temperature.The kidney tissues underwent overnight incubation at 4°C with primary antibodies (PLEKHA4,BD-PB3919, 1:300, Biodragon, Jiangsu, China; KLKI,YP-AB-02871, 1:200, UpingBio, Zhejiang, China; CLCNKB, DF9376, 1:150, Biodragon, Jiangsu, China) targeting the candidate biomarkers. Horseradish peroxidase (HRP)-conjugated antibodies were applied to the sections on the subsequent day. 3,3’-diaminobenzidine (DAB) (G1212-200T, Servicebio, Wuhan, China), a substrate specific to HRP, was used to highlight the stained areas in kidney tissue. Subsequently, counterstaining is performed using hematoxylin (G1004-100ML, Servicebio, Wuhan, China). Representative images were captured using an Olympus microscope, and ImageJ (NIH, USA) was employed to quantify the average optical density of the images to assess the expression levels of candidate biomarkers.

### 2.13 Immunofluorescent staining

Immunofluorescence staining was conducted on 5 μm-thick paraffin-embedded sections of mice kidney tissue. Following deparaffinization and antigen retrieval using EDTA (pH 9.0), the sections were blocked with goat serum and incubated overnight at 4°C with primary antibodies targeting SLC4A1 (A17391, 1:150, ABclonal, Wuhan, China) and CA II (EM1801-08, 1:150, HuaAn, Zhejiang, China). Subsequently, the sections were treated with iFluor™ 647-conjugated goat anti-rabbit IgG and iFluor™ 488-conjugated goat anti-mouse IgG (HA1125 and HA1123, 1:300, HuaAn) for one hour at room temperature. Nuclei were counterstained with DAPI, and imaging was performed using a Zeiss Axio-Imager A2 confocal microscope (Carl Zeiss, Jena, Germany).

### 2.14 Reverse-transcription polymerase chain reaction

In summary, total RNA was extracted from renal tissues using the Trizol method (15596026, Invitrogen, USA). Equivalent amounts of mRNA were reverse transcribed into cDNA utilizing the HiScript RT SuperMix kit (R122-01; Vazyme, China). Quantitative real-time PCR (qRT-PCR) was conducted with the ChamQ Universal SYBR qPCR Master Mix (Q711-02; Vazyme, China) on a Viia 7 quantitative real-time PCR instrument (Thermo-Fisher Scientific, USA). The PCR amplification protocol consisted of 35 cycles at 95°C for 30 seconds, 58°C for 30 seconds, and 72°C for 30 seconds. The following primers were employed: Aqp6 forward: GCCGTCATTGTTGGGAAGTTC and reverse: GGCTCCAGGTCTACCACTTTC; Kit forward: GAACAGGACCTCGGCTAACAA and reverse: CCTTTGCTCTGCTCCTGTACA;Slc4a1 forward: CCTCGTCCAATACATCTCCCG and reverse: CGTCATGGCAAGTAGGAAGGT. RT-PCR products were separated on a 1.5% agarose gel and visualized under UV light. The quantification of qRT-PCR was performed using the 2−ΔΔCt method and expressed as relative fold changes.

### 2.15 Patient samples

**Figure 1:**
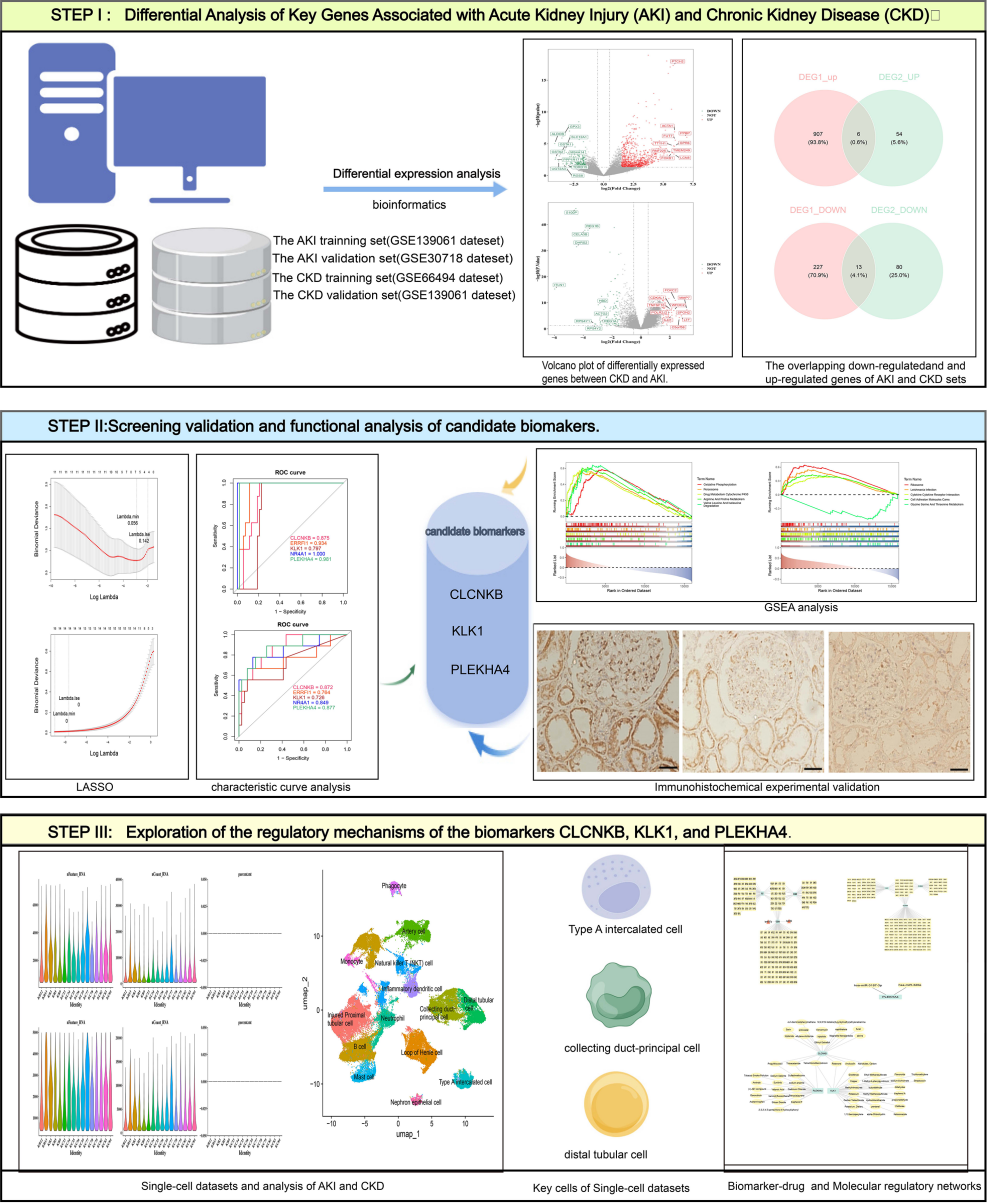
The overall workflow of this study. AKI, Acute kidney injury; CKD, Chronic kidney disease; GSEA, Gene set enrichment analysis.

To investigate the expression of candidate biomarkers in patients with AKI and CKD), we selected a cohort comprising five patients with AKI and five with CKD. Additionally, we included five patients diagnosed with renal malignancy, from whom normal renal tissue adjacent to the tumor was obtained during surgical procedures. Patients were identified as having AKI if they fulfilled any of these conditions: (a) a rise in serum creatinine (Scr) exceeding 26.5 μmol/L within 48 hours; (b) a 50% increase in Scr over the course of one week; or (c) urine output below 0.5 mL per kilogram per hour lasting over 6 hours. CKD patients were recognized by an estimated glomerular filtration rate (eGFR) under 60 mL/min/1.73 m².Approval for this study was granted by the ethics committee of The First Affiliated Hospital of Guangxi Medical University, with informed consent obtained from the patients (No. 2024-E0918).The data analysis design of this study was showed in **Figure 1**.

### 2.16 Statistical analysis

Bioinformatic analysis were performed in the R (v 4.2.2). GraphPad Prism statistical software was used for experiment statistical analyses in the study. We employed the unpaired t test to compare continuous variables between two groups. Values are shown as mean ± SEM, with statistical significance set at P<0.05.

## 3 Results

### 3.1 Candidate genes were ascertained

**Figure 2:**
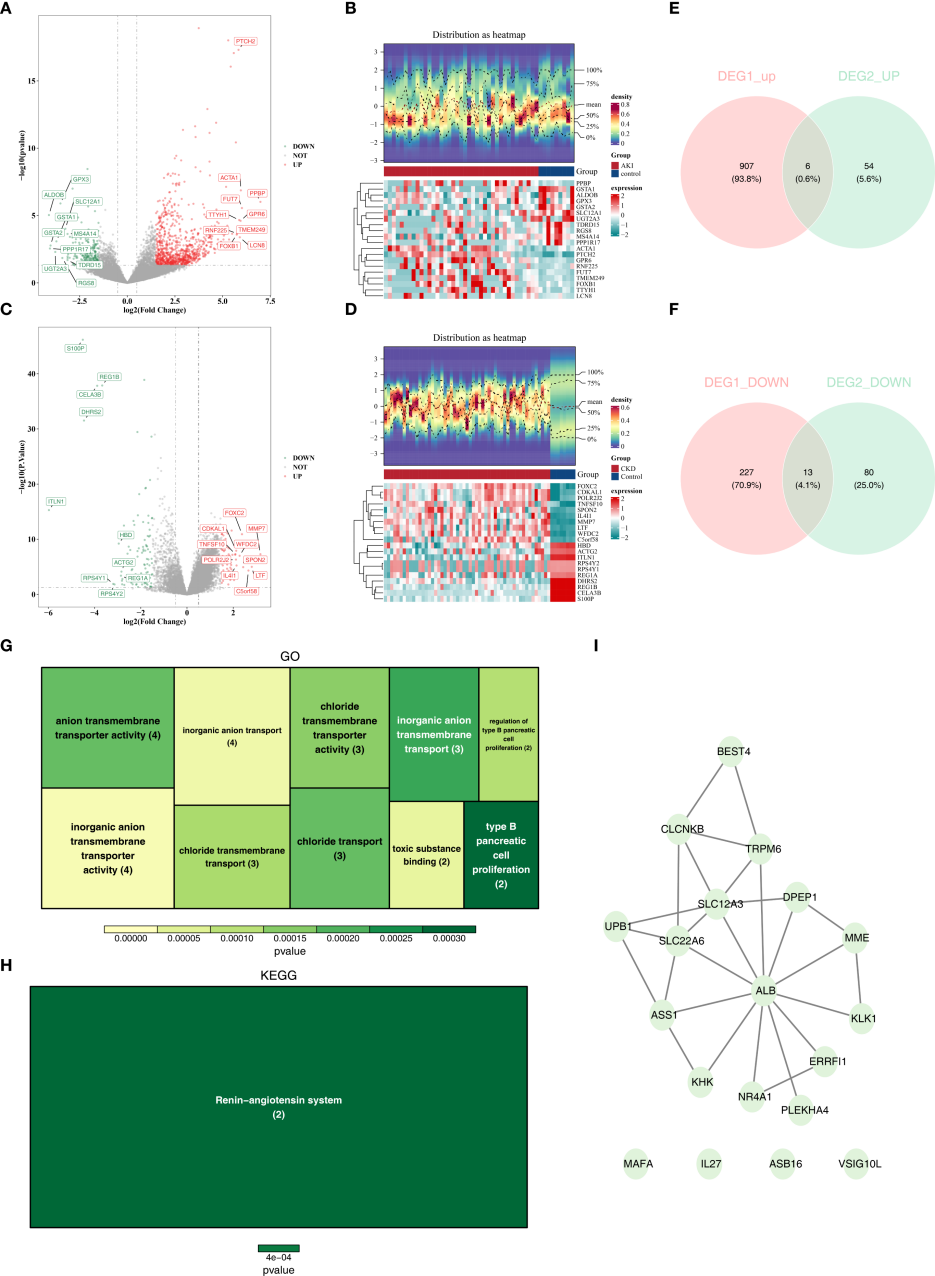
The different expressed genes(DEGs) of AKI and CKD sets and function and pathways of candidate genes. **(A, B)** The volcano plots of DEGs in AKI set. **(B)** The heatmaps of DEGs in CKD set. **(C)** The volcano plots of DEGs in AKI set. **(D)** The heatmaps of DEGs in CKD set. **(E)** The overlapping up-regulated genes of AKI and CKD sets. **(F)** The overlapping down-regulated genes of AKI and CKD sets. **(G)** GO analysis of candidate genes. **(H)** KEGG analysis of candidate genes. **(I)** PPI network of candidate genes. AKI, Acute kidney injury; CKD, Chronic kidney disease; DEGs, Differentially expressed genes1; GO, Gene Ontology (GO); KEGG, Kyoto Encyclopedia of the Genome; PPI, protein-protein interaction.

In the GSE139061 dataset, 1153 differentially expressed genes (DEGs1) were screened out, among which 913 were up-regulated and 240 were down-regulated. Similarly, in the GSE66494 dataset, 153 differentially expressed genes (DEGs2) were screened out, with 60 being up-regulated and 93 being down-regulated. The top 10 up- and down-regulated DEGs in both datasets and their expression profiles were labeled on the volcano plots and heatmaps respectively (**Figures 2A–D**). Subsequently, by overlapping the 913 up-regulated DEGs1 with the 60 up-regulated DEGs2, 6 common up-regulated genes were identified (**Figure 2E**). And by overlapping the 240 down-regulated DEGs1 with the 93 down-regulated DEGs2, 13 common down-regulated genes were obtained (**Figure 2F**). The 6 common up-regulated genes and the 13 common down-regulated genes were combined, and 19 candidate genes were determined. In conclusion, this analysis focused on the discovery of candidate genes that might play important roles in the progression of AKI and CKD.

### 3.2 Function and pathways of candidate genes were explored

Enrichment analyses of the 19 candidate genes showed that they were enriched in 22 GO entries, such as organic anion transport (**Figure 2G**; **Supplementary Table 3**), whereas KEGG analyses revealed that the candidate genes were significantly enriched in the Renin-angiotensin system (**Figure 2H**; **Supplementary Table 4**). In addition, in the constructed PPI network, genes such as ALB, SLC22A6 and SLC12A3 were highly associated with other genes (**Figure 2I**).

### 3.3 CLCNKB, KLK1, and PLEKHA4 were deemed as biomarkers

**Figure 3:**
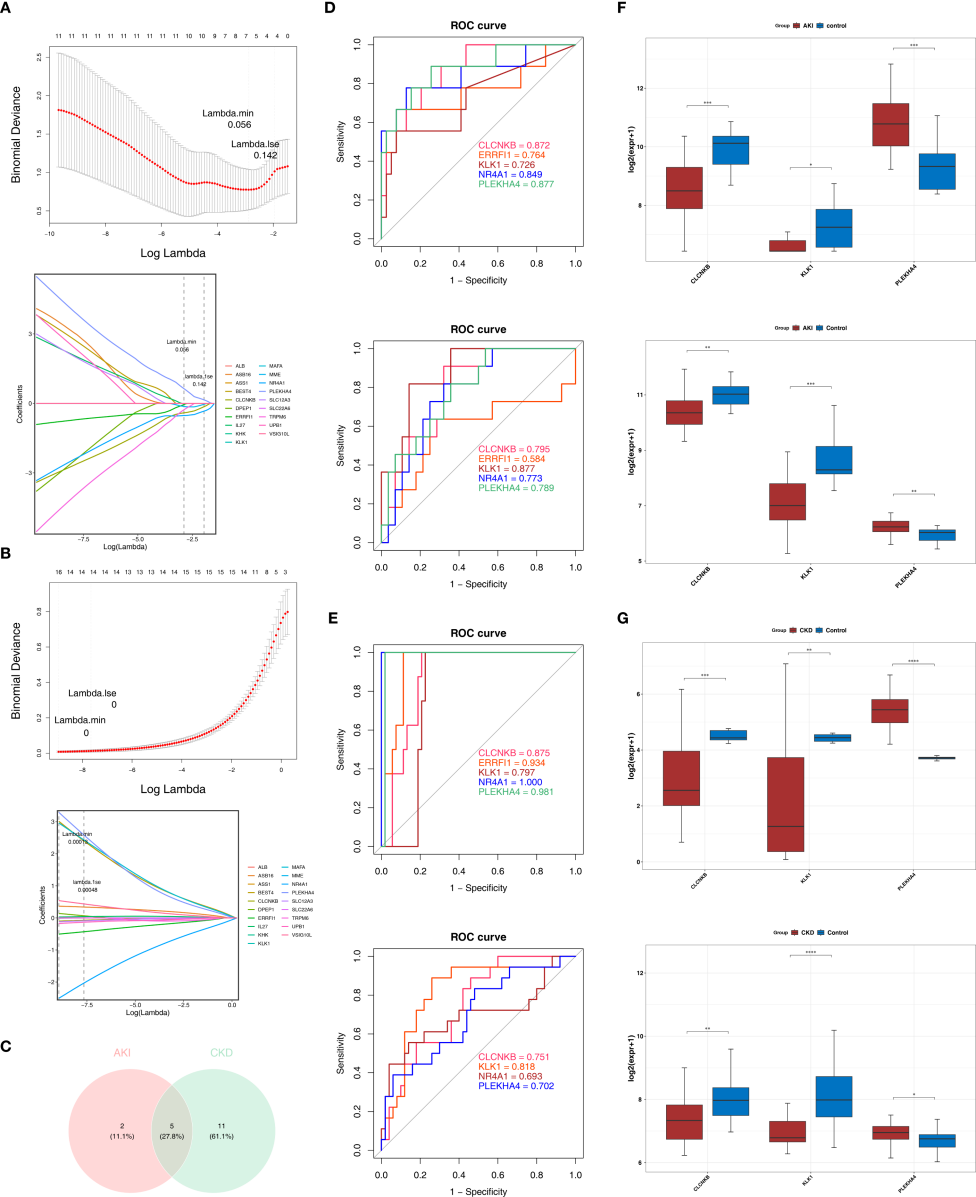
CLCNKB, KLK1 and PLEKHA4 were regarded as the candidate biomarkers and the GSEA analysis of candidate biomarkers. **(A, B)** LASSO regression analysis of AKI and CKD training sets. **(C)** Overlapping genes of AKI and CKD training sets. **(D, E)** Receiver operating characteristic curve analysis of AKI and CKD training sets and validation sets. **(F, G)** Expression trends of CLCNKB, KLK1 and PLEKHA4 in AKI and CKD training sets and validation sets. *p<0.05, **p<0.01, ***p<0.001, ****p<0.0001. AKI, Acute kidney injury; CKD, Chronic kidney disease; LASSO, Least absolute shrinkage and selection operator.

**Figure 4:**
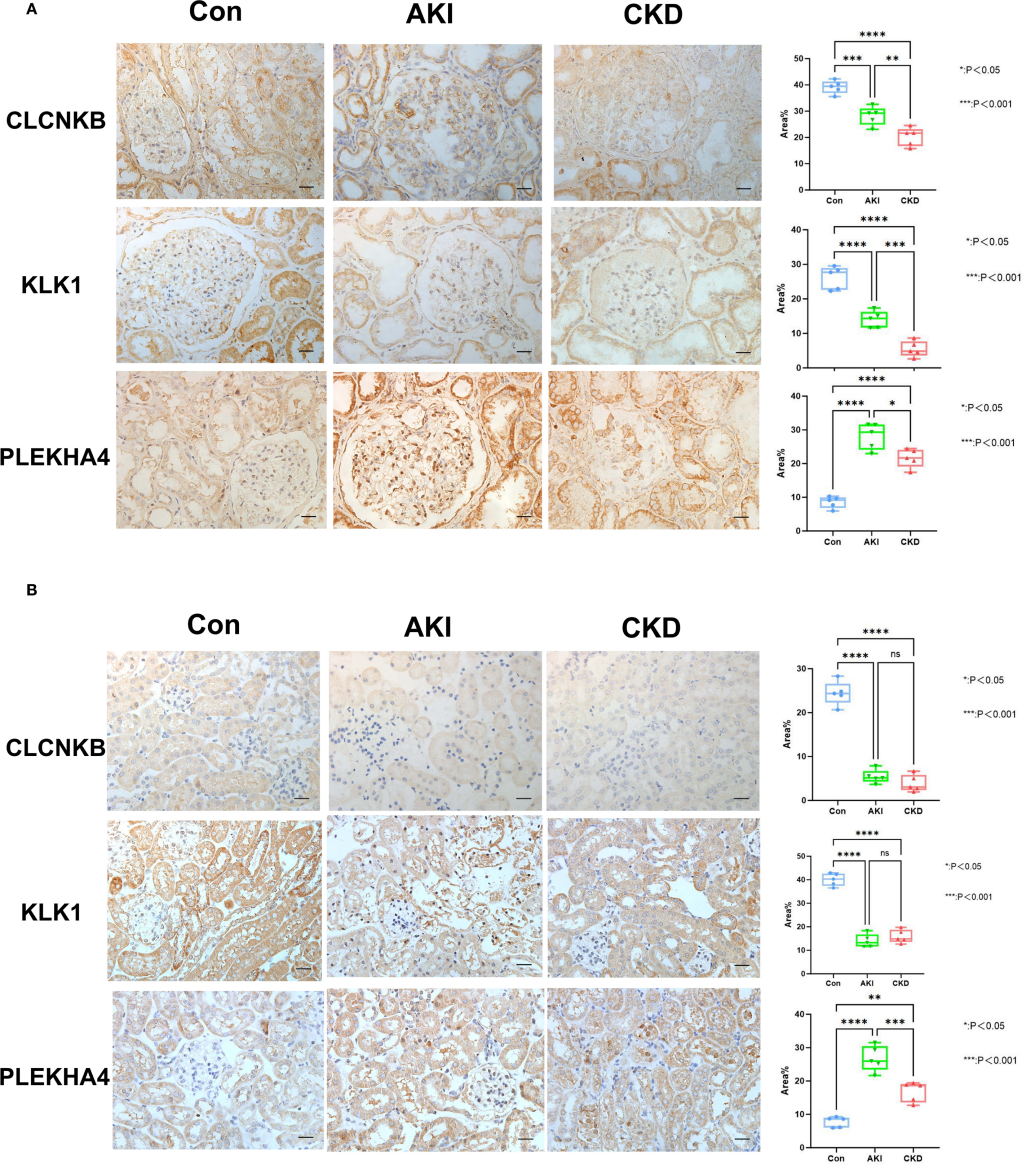
The verification of CLCNKB, KLK1 and PLEKHA4 in AKI and CKD samples. **(A)** The expression level of CLCNKB, KLK1 and PLEKHA4 in the kidneys of patients with AKI and CKD. **(B)** The expression level of CLCNKB, KLK1 and PLEKHA4 in the kidneys of AKI and CKD mice models. ns means not significant, *p<0.05, **p<0.01, ***p<0.001, ****p<0.0001.Bar:50um.

Based on the candidate genes, 7 potential feature genes in the AKI training set and 16 potential feature genes in the CKD training set were obtained respectively through the LASSO regression analysis (**Figures 3A, B**). Then, 5 feature genes were finally obtained by overlapping (**Figure 3C**). Subsequently, it was found in the AKI training set, validation set as well as the CKD training set and validation set that the AUC values of CLCNKB, KLK1 and PLEKHA4 were all greater than 0.7, and thus they could be regarded as the candidate biomarkers for this study (**Figures 3D, E**). Moreover, the expression analysis of the candidate biomarkers showed that the expression trends of CLCNKB, KLK1 and PLEKHA4 were consistent in the four datasets. Among them, CLCNKB and KLK1 were significantly down-regulated in AKI and CKD samples, while PLEKHA4 was significantly up-regulated (**Figures 3F, G**). Moreover, the expression trends of CLCNKB, KLK1 and PLEKHA4 in renal tissues of different groups of patients and different groups of mice models were consistent with our results (**Figure 4**).

### 3.4 Functional analysis helps explore potential mechanisms of AKI and CKD progression

**Figure 5:**
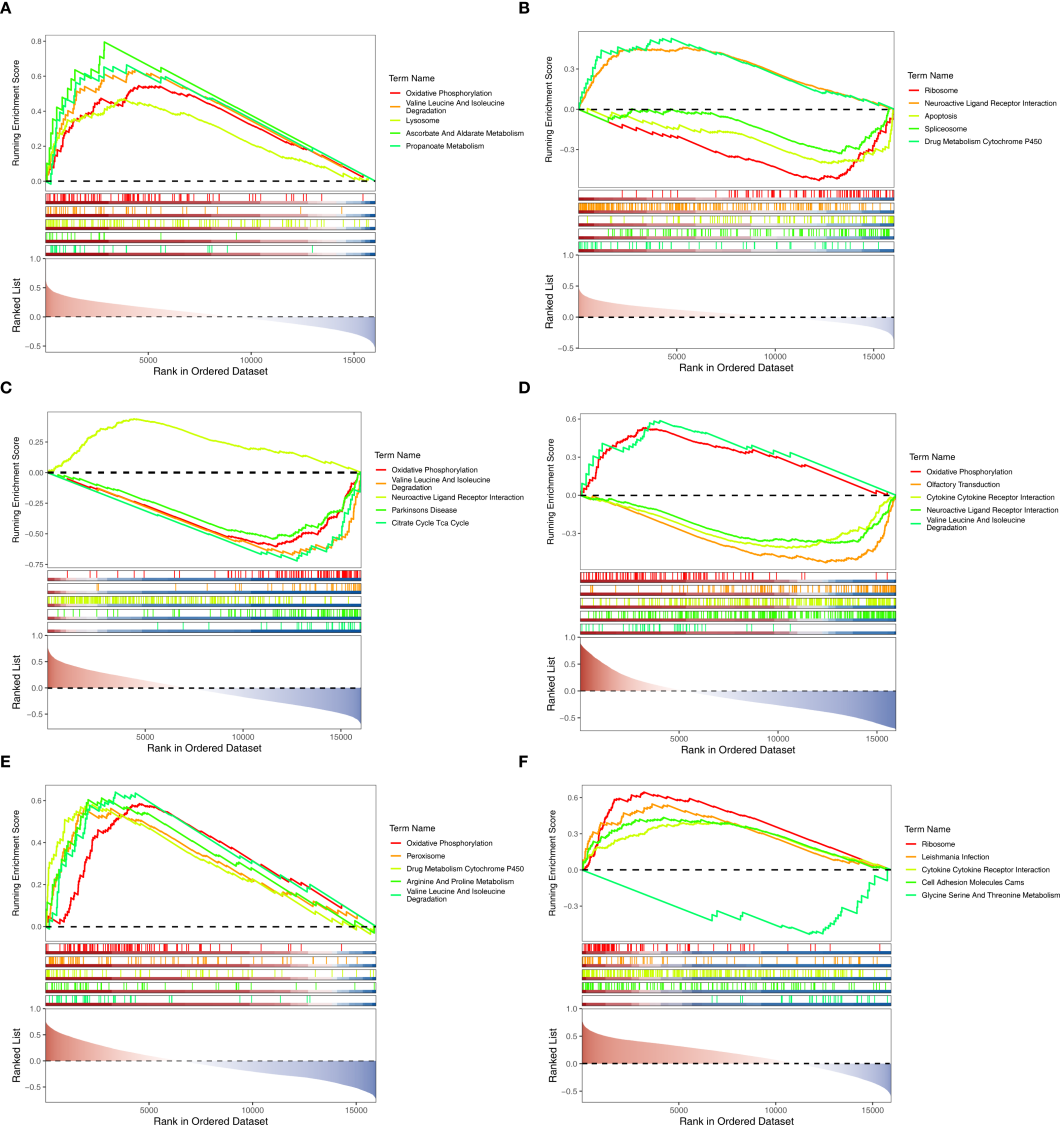
GSEA analysis of biomarkers. **(A–C)** GSEA analysis of CLCNKB, KLK1 and PLEKHA4 in AKI set. **(D–F)** GSEA analysis of CLCNKB, KLK1 and PLEKHA4 in CKD set. GSEA, Gene set enrichment analysis.

GSEA was performed on the GSE139061 and GSE6649 datasets to investigate the biological roles of the biomarkers. In the GSE139061 dataset of AKI, CLCNKB, KLK1 and PLEKHA4 were significantly enriched in 50, 15 and 51 pathways respectively (**Supplementary Tables 5**–**7**). It was worth noting that CLCNKB and PLEKHA4 were co-enriched in the valine leucine and isoleucine degradation pathways and oxidative phosphorylation, and KLK1 and PLEKHA4 were co-enriched in the neuroactive ligand receptor interaction pathway (**Figures 5A–C**). In the GSE6649 dataset of CKD, CLCNKB, KLK1 and PLEKHA4 were significantly enriched in 39, 60 and 44 pathways respectively (**Supplementary Tables 8**–**10**). Among them, CLCNKB and KLK1 were jointly enriched in the oxidative phosphorylation and valine, leucine, and isoleucine degradation pathways, as well as in the cytokine-cytokine receptor interaction pathway (**Figures 5D–F**).

### 3.5 GYG1 and PPP1R3D were associated with immune infiltrating cells

**Figure 6:**
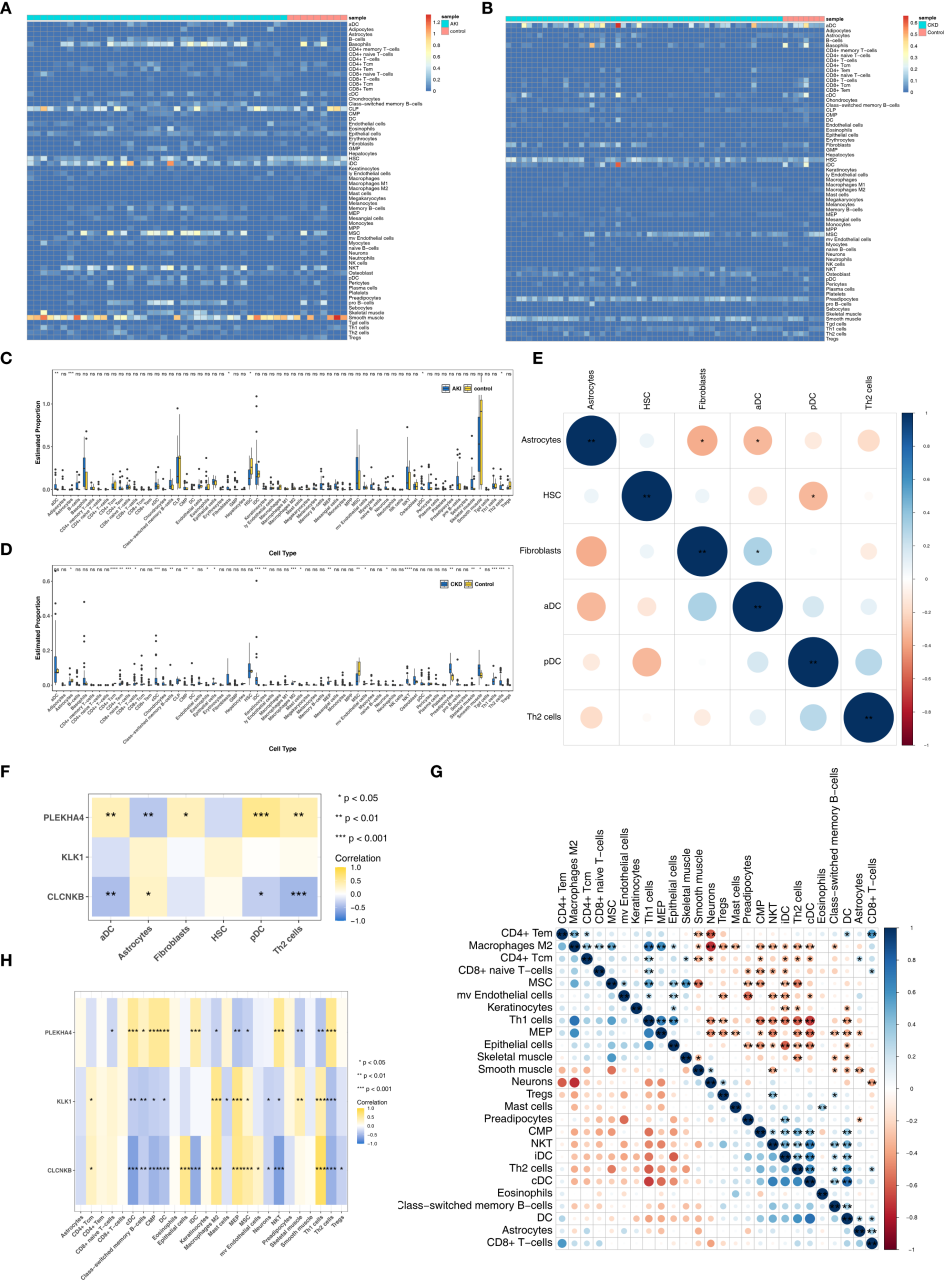
Immune infiltrating cells of AKI and CKD. **(A, B)** Infiltration levels of 64 immune cells in AKI and CKD set. **(C)** The different immune cells in AKI and control samples. **(D)** The different immune cells in CKD and control samples. **(E)** The correlation of different immune cells in AKI set. **(F)** Correlation of immune cells and candidate biomarkers in AKI set. **(G)** The correlation of different immune cells in CKD set. **(H)** Correlation of immune cells and candidate biomarkers in CKD set. AKI, Acute kidney injury; CKD, Chronic kidney disease. *p<0.05, **p<0.01, ***p<0.001, ****p<0.0001.

**Figures 6A, B** illustrated the infiltration levels of 64 immune cells in AKI versus control samples, and CKD versus control samples, respectively. Among them, the infiltration levels of 6 types of immune cells (differential immune cells 1) were significantly different in AKI and control samples (**Figure 6C**), and the infiltration levels of 26 types of immune cells (differential immune cells 2) were significantly different in CKD and control samples (**Figure 6D**), and the common differential immune cells included Astrocytes, Th2 cells. Furthermore, among the differential immune cells 1 in AKI, Fibroblasts had the most significant positive correlation with aDC (cor = 0.32), and Astrocytes had the most significant negative correlation with Fibroblasts (cor = -0.36) (**Figure 6E**; **Supplementary Table 11**). Whereas PLEKHA4 had the strongest positive relationship with pDC (cor = 0.58) and the strongest negative relationship with Astrocytes (cor = -0.38), CLCNKB had the strongest positive relationship with Astrocytes (cor = 0.30) and the strongest negative relationship with Th2 cells (cor = -0.49), but KLK1 was significantly correlated with Differential Immune Cells 1 (**Figure 6F**; **Supplementary Table 12**). Subsequently, among the differential immune cells 2 in CKD, cDC had the most significant positive correlation with DC (cor = 0.75) and the highest positive association with Macrophages M2 and Neurons (cor = -0.74) (**Figure 6G**; **Supplementary Table 13**). In contrast, PLEKHA4 had the greatest positive connection with Th2 cells (cor = 0.58) and the greatest negative linkage with MEP (cor = -0.40), CLCNKB had the most significant positive correlation with Th1 cells (cor = 0.79) and the strongest inverse relationship with NKT (cor = -0.67), and KLK1 had the greatest positive connection with MEP (cor = 0.62) and the most prominent negative connection with Th2 cells (cor = -0.43) (**Figure 6H**; **Supplementary Table 14**).

### 3.6 Molecular regulatory networks probe regulatory mechanisms of biomarkers

**Figure 7:**
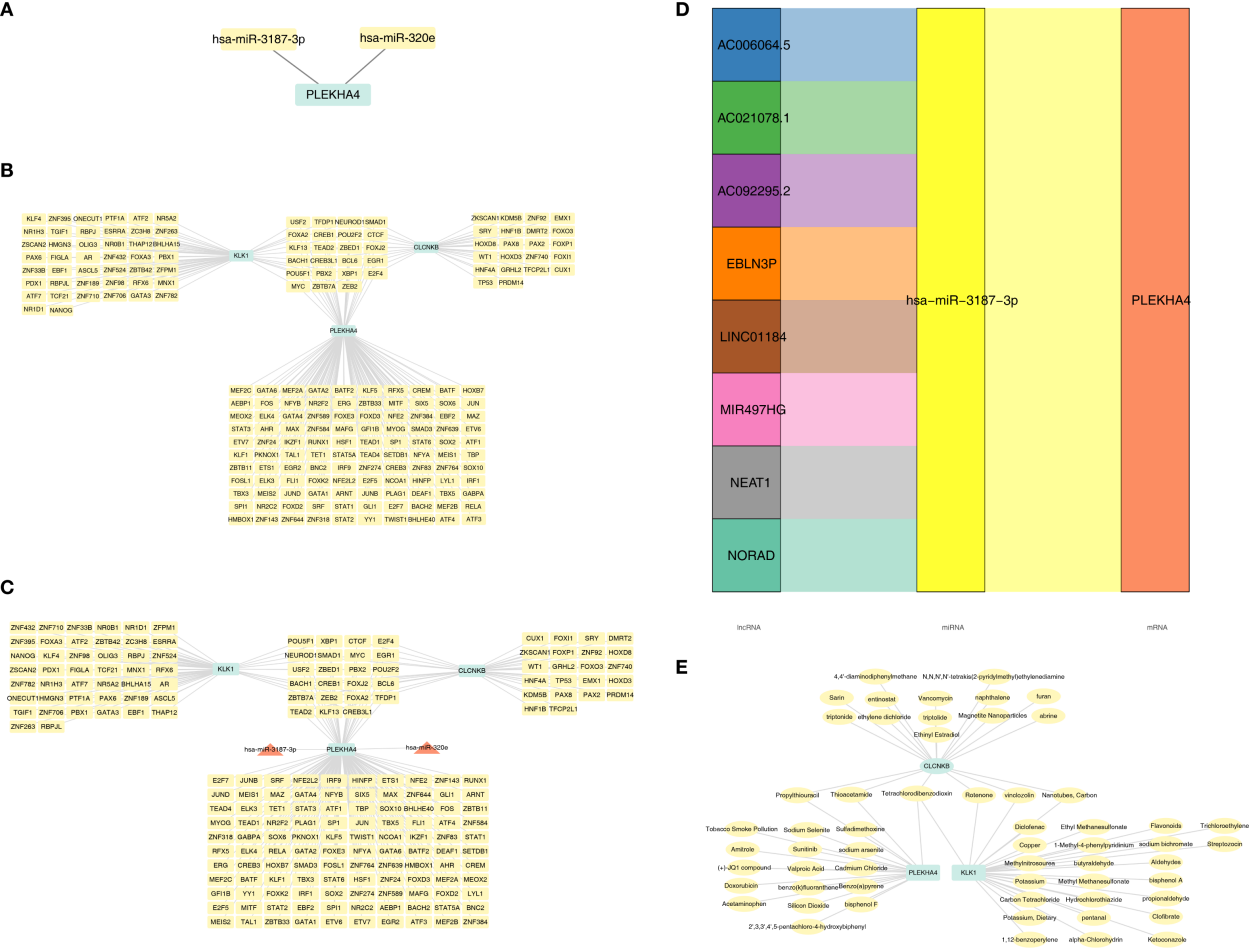
Molecular regulatory networks and biomarker-drug network of candidate biomarkers. **(A)** The miRNAs were predicted for candidate biomarkers. **(B)** TF-mRNA networks of candidate biomarkers. **(C)** miRNA-mRNA-TF network of candidate biomarkers. **(D)** lncRNA-miRNA-mRNA network of candidate biomarkers. **(E)** Biomarker-drug network of CLCNKB, KLK1 and PLEKHA4. TF, Transcription factors.

Initially, merely 2 miRNAs were predicted for PLEKHA4, whereas no miRNAs could be predicted for CLCNKB and KLK1 (**Figure 7A**). Subsequently, the TF-mRNA networks consisting of 133, 34 and 56 TFs corresponding to PLEKHA4, CLCNKB and KLK1 respectively were acquired from the ChEA3 database (**Figure 7B**). Then, a miRNA-mRNA-TF network was established by integrating the 2 miRNAs (**Figure 7C**). Eventually, 8 lncRNAs upstream of miRNAs were predicted and a lncRNA-miRNA-mRNA network was constructe, such as EBLN3P-hsa-miR-3187-3p-PLEKHA4 (**Figure 7D**). In a nutshell, this analysis centered around the finding that PLEKHA4 was likely to play a more crucial role in the progression of AKI and CKD.

### 3.7 CLCNKB, PLEKHA4 and KLK1 were simultaneously targeted by Tetrachlorodibenzodioxin

Drugs were screened for activation of CLCNKB and KLK1, which are down-regulated in expression, and inhibition of PLEKHA4, which is up-regulated in expression, including 27 drugs targeting KLK1, 19 drugs targeting CLCNKB and 19 drugs targeting PLEKHA4 (**Supplementary Tables 15**–**17**). A biomarker-drug network was constructed accordingly (**Figure 7E**). It was noteworthy that CLCNKB, PLEKHA4 and KLK1 were simultaneously targeted by Tetrachlorodibenzodioxin.

### 3.8 Annotation in AKI and CKD yielded 14 and 13 cell types, respectively

**Figure 8:**
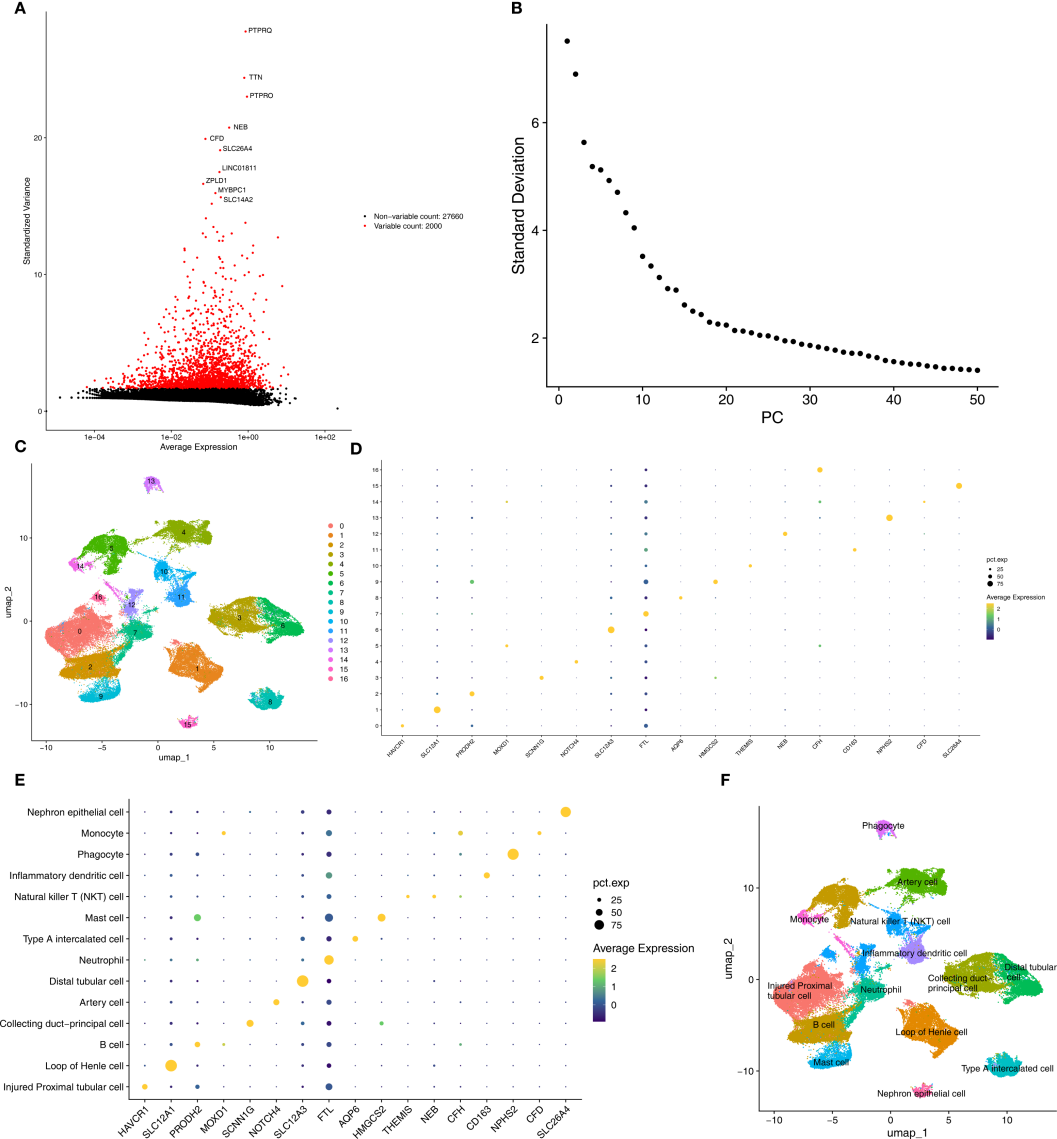
Annotated cell types in AKI groups. **(A, B)** Top variable genes and PCs were applied to UMAP clustering. **(C)** Different cell cluster of high-quality cells of AKI single-cell dataset. **(D, E)** Marker genes identified different cell clusters. **(F)** 14 cell types were determined in AKI single-cell dataset. AKI, Acute kidney injury; UMAP, Uniform manifold approximation and projection.

**Figure 9:**
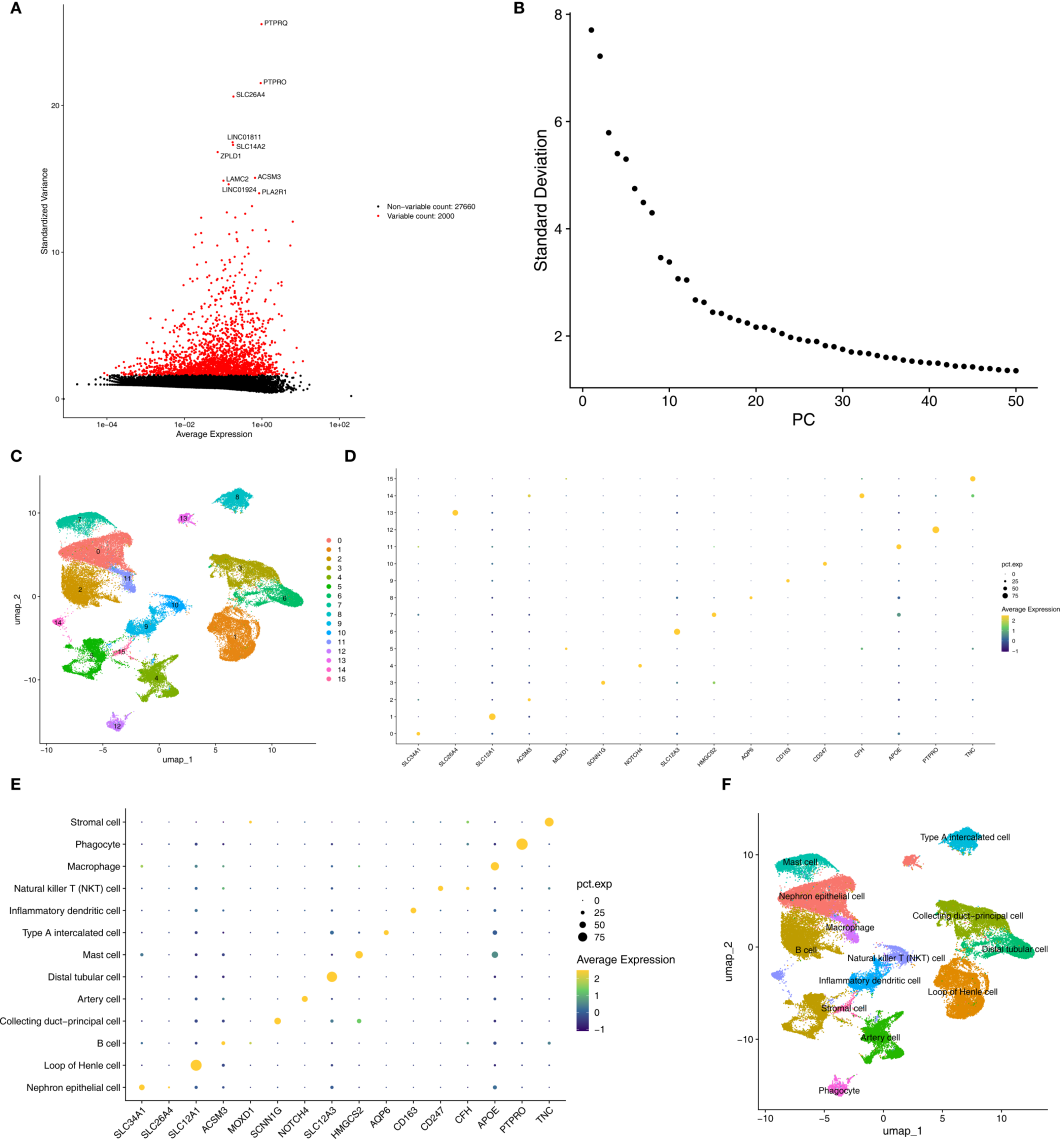
Annotated cell types in CKD groups. **(A, B)** Top variable genes and PCs were applied to UMAP clustering. **(C)** Different cell cluster of high-quality cells of CKD single-cell dataset. **(D, E)** Marker genes identified different cell clusters. **(F)** 13 cell types were determined in AKI single-cell dataset. CKD, Chronic kidney disease; UMAP, Uniform manifold approximation and projection.

In the AKI single-cell dataset, a total of 78,791 cells were retained after quality control (**Supplementary Figure S1**). Subsequently, the top 2,000 highly variable genes and the top 30 PCs were applied to UMAP clustering (**Figures 8A, B**). All high-quality cells were divided into 17 different cell clusters (**Figure 8C**). In addition, marker genes had high specificity in different cell clusters (**Figures 8D, E**). The cell clusters were annotated and 14 cell types were determined, such as Injured Proximal tubular cell and Loop of Henle cell (**Figure 8F**). Subsequently, in the CKD single-cell dataset, a total of 58,561 cells were retained after quality control (**Supplementary Figure S2**). Next, the top 2,000 highly variable genes and the top 30 PCs were applied to UMAP clustering (**Figures 9A, B**). All high-quality cells were divided into 16 different cell clusters (**Figure 9C**). Moreover, marker genes also had high specificity in different cell clusters (**Figures 9D, E**). The cell clusters were annotated and 13 cell types were determined, such as Nephron epithelial cell and Loop of Henle cell (**Figure 9F**).

### 3.9 Type A intercalated cell and collecting duct-principal cell identified as key cells

**Figure 10:**
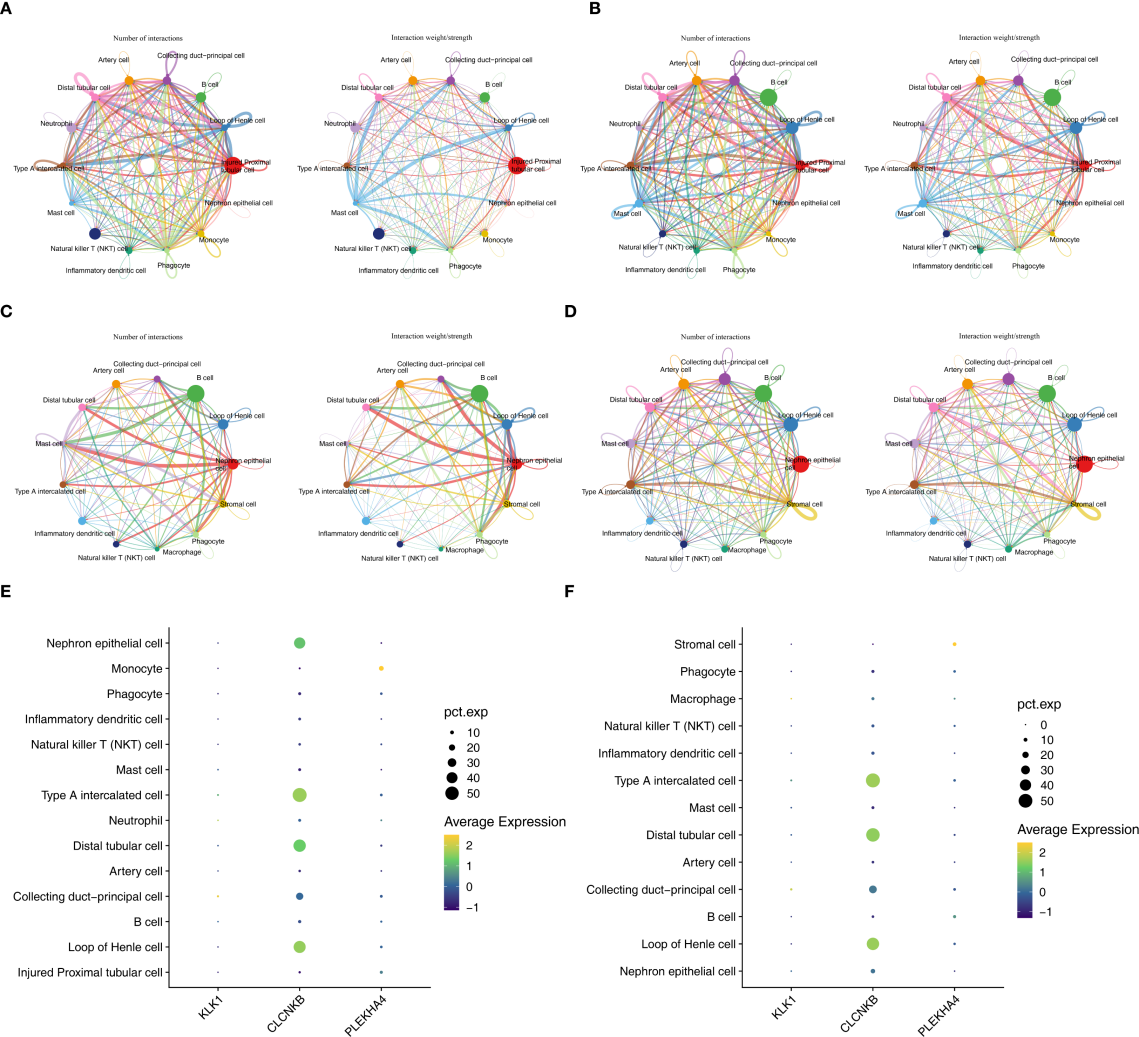
Key cells and expression trends of candidate biomarkers during development of key cell sub-populations of AKI and CKD single-cell datasets. **(A, B)** The cell interactions of AKI and control samples. **(C, D)** The cell interactions of CKD and control samples. **(E, F)** Cells with high expression of candidate biomarkers in AKI and CKD datasets.

**Figure 11:**
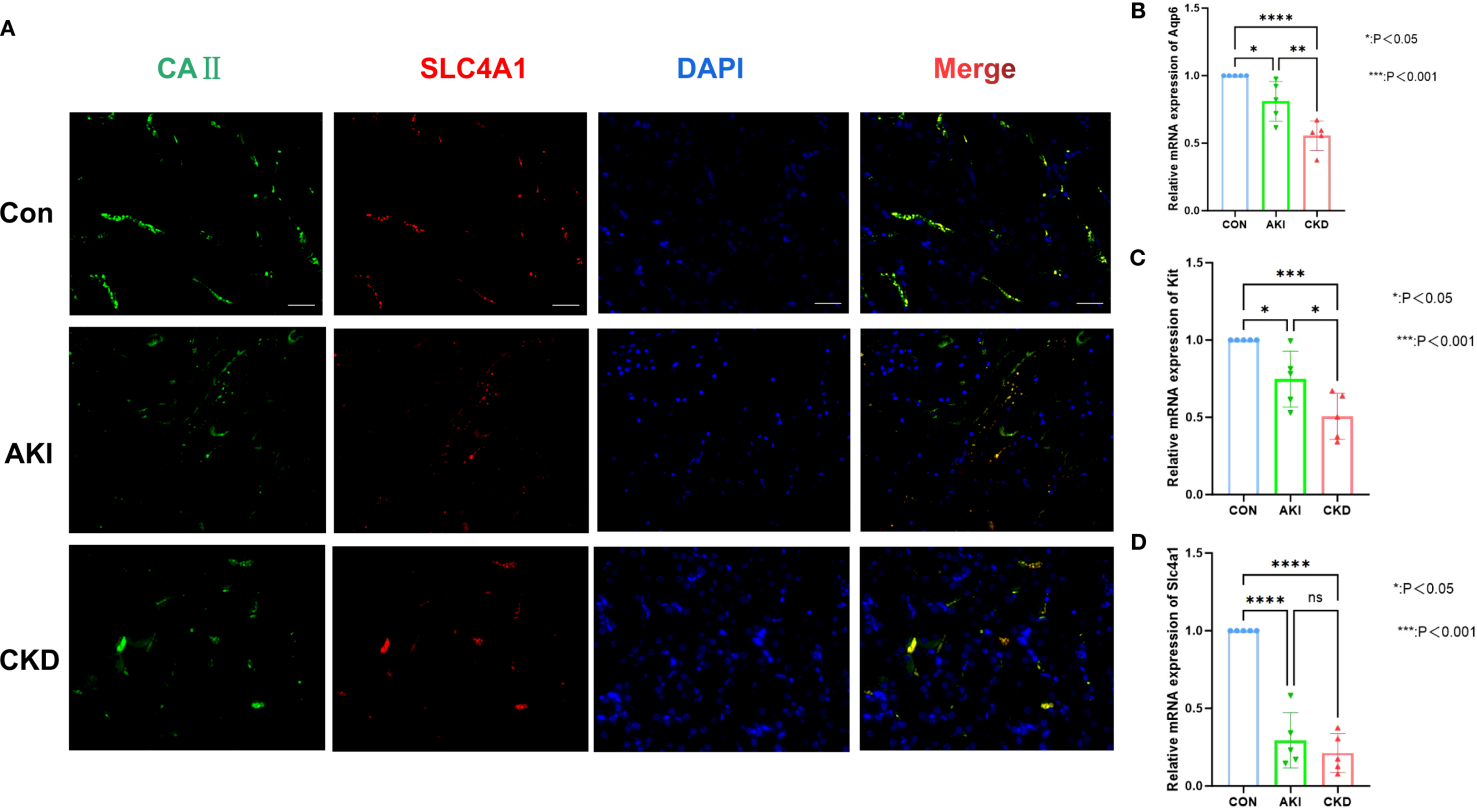
Abundance of type A intercalated cells in AKI/CKD mice. **(A)** Immunostaining of intercalated cell maker CAII (green) and the type A intercalated cell maker SLC4A1 (red) in renal collecting duct of AKI/CKD mice. **(B–D)** Relative expression level of type A intercalated cell maker (Aqp6, Kit and Slc4a1) in the kidneys of AKI/CKD mice.ns means not significant, *p<0.05, **p<0.01, ***p<0.001, ****p<0.0001.Bar:50um.

In the AKI single-cell dataset, the injured proximal tubular cells in AKI samples had a relatively large number of interactions and a relatively high intensity with other cells (**Figure 10A**), while in the control samples, B cells had a relatively large number of interactions and a relatively high intensity with other cells (**Figure 10B**). Interestingly, in the CKD single-cell dataset, Nephron epithelial cells and B cells had a relatively large number of interactions and a relatively high intensity with other cells both in CKD and control samples (**Figures 10C, D**). In addition, KLK1 and CLCNKB had relatively high expression levels in Type A intercalated cells and Collecting duct-principal cells in both single-cell datasets (**Figures 10E, F**). To evaluate the abundance of type A intercalated cells in kidney disease, immunofluorescence staining was performed on kidney sections of AKI/CKD mice. Consistent with the reduced expression of SLC4A1 (a specific marker for this cell type) in diseased kidneys (**Figure 11A**), the expressions of additional markers (Aqp6, Kit and Slc4a1) were also significantly downregulated (**Figures 11B–D**), confirming the loss of type A intercalated cells in AKI/CKD. Consequently, Type A intercalated cells could have been part of the disease’s development.

### 3.10 CLCNKB, KLK1, and PLEKHA4 expression changes during development of key cell subpopulations

**Figure 12:**
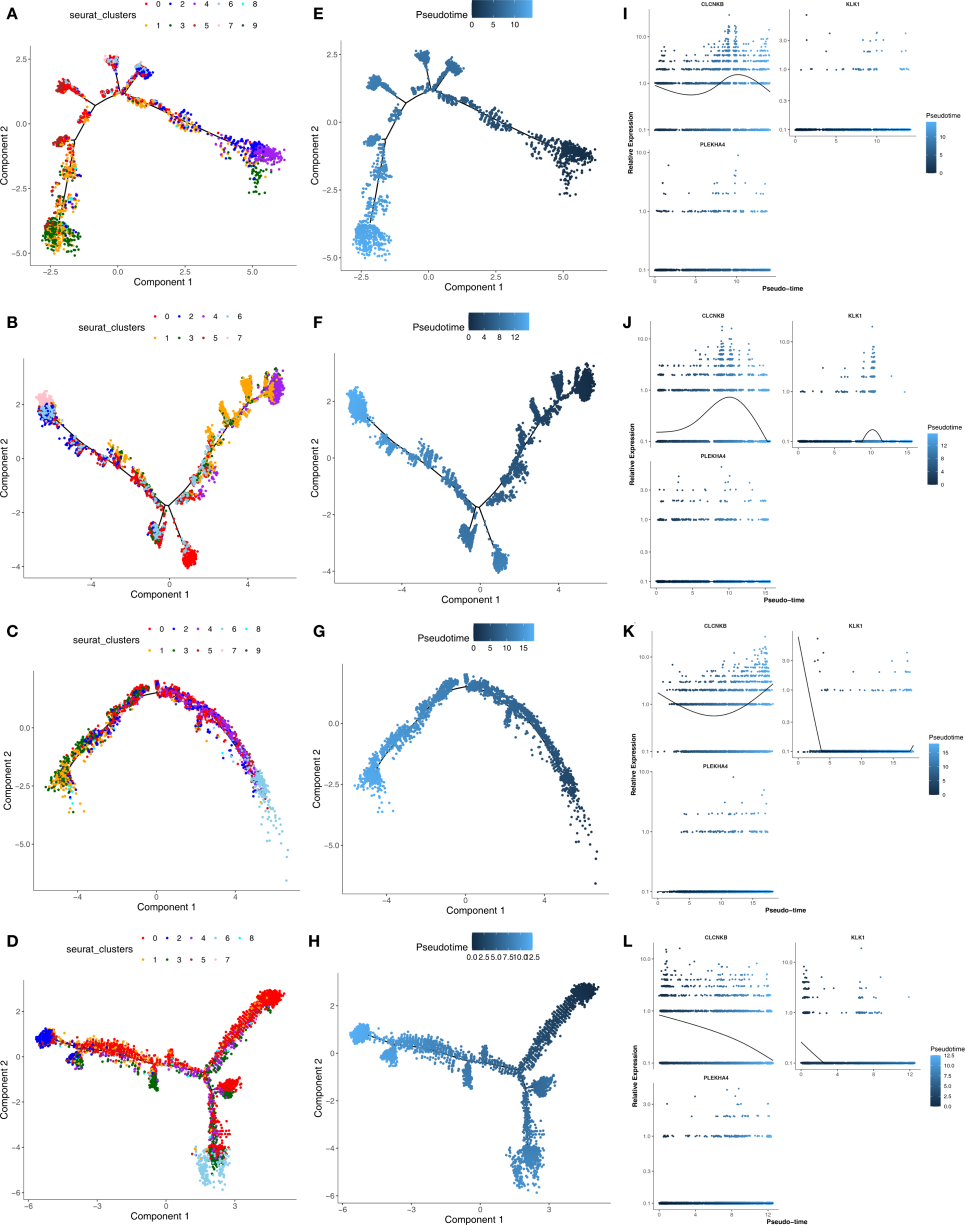
**(A, B)** The subgroups of Type A intercalated cell and Collecting duct-principal cell in AKI single-cell dataset. **(C, D)** The subgroups of Type A intercalated cell and Collecting duct-principal cell in CKD single-cell dataset. **(E, F)** Different states of Type A intercalated cell and Collecting duct-principal cell in AKI single-cell dataset. **(G, H)** Different states of Type A intercalated cell and Collecting duct-principal cell in CKD single-cell dataset. **(I, J)** Expression trends of CLCNKB、KLK1 and PLEKHA4 in Type A intercalated cell and Collecting duct-principal cell in AKI single-cell dataset. **(K, L)** Expression trends of CLCNKB、KLK1 and PLEKHA4 in Type A intercalated cell and Collecting duct-principal cell in CKD single-cell dataset. AKI, Acute kidney injury; CKD, Chronic kidney disease.

Secondary dimensionality reduction clustering analysis was performed on Type A intercalated cell and Collecting duct-principal cell. It was found that Type A intercalated cell and Collecting duct-principal cell were divided into 10 and 8 subgroups respectively in the AKI single-cell dataset (**Figures 12A, B**). Whereas in the CKD single-cell dataset, Type A intercalated cell was divided into 10 subgroups and Collecting duct-principal cell was divided into 9 subgroups (**Figures 12C, D**). Subsequently, the different subgroups within Type A intercalated cell and Collecting duct-principal cell were arranged on the developmental trajectory according to the differentiation time. A darker blue indicates earlier cell differentiation. In addition, after different cell subgroups were mapped to the pseudo-time trajectory plot, it was found that they exhibited different differentiation states. In the AKI single-cell dataset, Type A intercalated cell had 10 differentiation states, with State 4 being the earliest and most specific in differentiation. Collecting duct-principal cell had 8 differentiation states, and State 4 was also the earliest and most specific (**Figures 12E, F**). In the CKD single-cell dataset, Type A intercalated cell also had 10 differentiation states, with State 9 being the earliest in differentiation. Collecting duct-principal cell had 8 differentiation states, and State 0 was the earliest and most specific (**Figures 12G, H**). In the AKI single-cell dataset, with the differentiation of Type A intercalated cells, the expressions of KLK1 and PLEKHA4 had no significant changes. The expression of CLCNKB showed a trend of first decreasing, then increasing and finally decreasing again (**Figures 12I**). With the development of Collecting duct-principal cells, PLEKHA4 had no significant change. The expression of CLCNKB showed a trend of first increasing and then decreasing, and the expression of KLK1 showed a trend of first remaining unchanged, then increasing, then decreasing and finally remaining unchanged (**Figures 12J**) In the CKD single-cell dataset, with the development of Type A intercalated cells, PLEKHA4 had no significant change. The expression of CLCNKB showed a trend of first decreasing and then increasing, and the expression of KLK1 showed a trend of first decreasing, then remaining unchanged and finally increasing (**Figures 12K**). With the development of Collecting duct-principal cells, PLEKHA4 had no significant change. CLCNKB expression consistently declined, while KLK1 expression initially decreased and then stabilized (**Figures 12L**).

## 4 Discussion

AKI is marked by a swift reduction in kidney function over a brief period, and the transition from AKI to CKD is a widely recognized clinical occurrence. Our study identified three biomarkers (CLCNKB, KLK1, and PLEKHA4) through a combination of machine learning algorithms, ROC curve analysis, and expression validation. The potential mechanisms associated with these biomarkers in AKI and CKD were explored through enrichment analysis, regulatory network construction, immune infiltration analysis, and drug target prediction. By integrating single-cell data, we identified key cell types and investigated the expression of these biomarkers at the cellular level. Thus, our investigation discovered some new perspectives on the potential pathogenesis and progression of AKI to CKD, which might provide therapeutic targets to avert the transition.

CLCNKB is responsible for encoding the CLC-Kb protein, a component of the CLC chloride channel family, that helps with chloride ion reabsorption in the renal tubules (32, 33).Genetic mutations in CLCNKB can impair the glycosylation of the CLC-Kb protein, compromising its functionality and resulting in reduced uptake of sodium and chloride ions in the kidney tubules (34). Mutations in the CLCNKB gene are notably linked to Bartter syndrome type III, a rare hereditary renal tubular disorder characterized by salt loss and electrolyte imbalances, frequently culminating in CKD (35, 36).Our study demonstrated a significant down-regulation of CLCNKB in renal tissue samples from patients with both AKI and CKD, aligning with the loss-of-function effect indicated by the aforementioned genetic evidence. Moreover, this substantial loss of sodium and chloride ions triggers the activation of the renin – angiotensin – aldosterone system (RAAS), which may exacerbate kidney injury in AKI and facilitate the development to CKD (37).During the acute phase, persistent activation of the RAAS may exacerbate AKI-induced renal damage by promoting vasoconstriction and inflammatory responses (37). Over the long term, this mechanism is pivotal in driving renal fibrosis and glomerulosclerosis, thereby expediting the progression from AKI to CKD (38). Functional enrichment analysis corroborated this mechanism. Furthermore, CLCNKB was found to be significantly associated with metabolic pathways, such as oxidative phosphorylation and branched-chain amino acid degradation, suggesting that its down-regulation may also be implicated in energy metabolism disorders within renal tubular cells, collectively facilitating the chronic progression of the disease.

KLK1 is a serine protease that plays a pivotal role in the kininase-kinin system (KKS) by breaking down low molecular weight kininogen to yield bradykinin (BK) (39). The KKS is intricately associated with several physiological processes, including inflammation, coagulation, pain, and increased vascular permeability, all of which are mediated by kinin production (40). KLK1 is notably involved in the signaling pathways of the B1 receptor for bradykinin (B1R) and the B2 receptor for bradykinin (B2R), thereby triggering a series of physiological responses that produce anti-apoptotic, anti-inflammatory, anti-fibrotic, and antioxidant effects. These actions collectively contribute to tissue protection, underscoring the multifaceted beneficial roles of KLK1 in maintaining tissue homeostasis (41). Furthermore, previous research has demonstrated that Klk1 ameliorates lupus nephritis in murine models (42, 43). The functional enrichment analysis conducted in this study revealed a significant association between the down-regulation of KLK1 expression and the neuroactive ligand-receptor interaction and cytokine-cytokine receptor interaction signaling pathways. This suggests that reduced KLK1 expression may compromise the protective function of renal tubular cells by disrupting bradykinin signaling and exacerbating the inflammatory microenvironment.Consequently, the absence of KLK1 may be implicated in the development from AKI to CKD.

PLEKHA4 encodes a protein characterized by a Pleckstrin homology domain near its N-terminus and has an important function in cancer biology, particularly in gliomas. Furthermore, PLEKHA4 regulates the Wnt/β-catenin signaling pathway. *In vitro* downregulation of PLEKHA4 resulted in decreased dishevelled protein levels and a later diminishment of Wnt/β-catenin signaling (44).Conversely, overexpression of PLEKHA4 activated the Wnt/β-catenin pathway, facilitating the transfer of β-catenin to the nucleus and promoting signaling activity (45). The Wnt/β-catenin pathway, a developmental signaling cascade typically inactive in the adult kidney, becomes reactivated in various renal pathologies and plays a pivotal role in the pathogenesis of CKD (46, 47). Continuous activation of the Wnt/β-catenin signaling pathway has been linked to the advancement of kidney fibrosis, podocyte injury, and proteinuria in CKD (48–50), as well as contributing to AKI and sustained tissue damage in cystic kidney disease (51, 52). Furthermore, molecular regulatory networks suggest that PLEKHA4 may play a significant role in the progression from AKI to CKD. Consequently, the overexpression of PLEKHA4 could potentially exacerbate kidney damage in AKI and expedite the progression from AKI to CKD, warranting further investigation into the underlying mechanisms.

Our study indicates that during the progression from AKI to CKD, the oxidative phosphorylation pathway and the degradation pathways of valine, leucine, and isoleucine are pivotal. Following acute kidney injury, the renal repair process is often maladaptive, resulting in the dedifferentiation of tubular cells and the intensification of inflammatory responses. This maladaptive repair mechanism is intricately linked to the dysregulation of oxidative phosphorylation, which subsequently impacts long-term kidney function (53, 54). Research has demonstrated a significant association between valine degradation disorder and renal fibrosis, a critical pathological feature of CKD (55).Similarly, amino acid metabolism assumes a pivotal role in CKD (56). Amino acids can influence renal lesions and fibrosis through the aryl hydrocarbon receptor (AhR) signaling pathway (57). Certain amino acids, such as taurine, exhibit renoprotective properties by safeguarding the mitochondrial membrane and inhibiting cell apoptosis, thereby mitigating structural damage to the renal cortex (58, 59). The significance of amino acid metabolism in disease mechanisms positions it as a potential target for the early diagnosis and treatment of CKD (60). In CKD, amino acid metabolism is markedly disrupted, typically evidenced by elevated levels of arginine and citrulline and a decreased ornithine/citrulline ratio, indicating that citrulline may serve as a potent biomarker of renal metabolism (61). This metabolic disturbance interacts with systemic inflammation and metabolic acidosis, disrupting amino acid and protein homeostasis. As CKD progresses, glomerular filtration and renal tubular reabsorption functions are further compromised, exacerbating amino acid depletion and proteinuria, thereby perpetuating a detrimental cycle (62). Therefore, interventions targeting oxidative phosphorylation pathways and amino acid metabolism may offer advanced therapeutic techniques to decelerate the progression from AKI to CKD.

During the transition from AKI to CKD, various cell types including fibroblasts, Th2 cells, astrocytes, DCs, and M2 macrophages, play pivotal roles, aligning with our findings. Studies indicate that fibroblasts differentiate into myofibroblasts following kidney injury, thereby promoting extracellular matrix accumulation and contributing to renal fibrosis (63). Moreover, M2 macrophages exhibit a dual role in this process, engaging in tissue repair while potentially facilitating fibrosis progression in chronic inflammation (64). Post-AKI, Th2 cell activation may mitigate inflammatory responses and promote renal repair.However, an excessive Th2 cell response can also exacerbate fibrosis (65). In addition, suppressing PLEKHA4 might obstruct the M2 polarization process in macrophages (66). Thus, the positive association of Th2 cells with PLEKHA4 may expedite the progression of kidney fibrosis. Dendritic cells modulate T cell activation and differentiation through antigen presentation and cytokine secretion, thereby impacting the inflammatory and reparative mechanisms of kidney (67). Meanwhile, astrocytes are integral to the neuroimmune regulation of the kidney, potentially influencing the inflammatory response and fibrotic processes via the release of neurotransmitters and cytokines (68). It is noteworthy that interstitial cells, as a crucial component of the renal microenvironment, play a significant role in renal inflammation. They amplify local inflammatory signals by releasing proinflammatory factors, thereby inducing increased apoptosis of renal parenchymal cells and exacerbating renal fibrosis through the promotion of myofibroblast activation and extracellular matrix deposition (69). This process is intricately linked to abnormal oxidative stress, which not only results from the inflammatory response but also exacerbates inflammation and apoptosis by impairing mitochondrial function and activating the NF-κB and Nrf2 signaling pathways (70–72). These pathways are central to the regulation of apoptosis, inflammation (73), and oxidative stress in kidney diseases and are pivotal in determining the progression and outcomes of CKD (74, 75). Furthermore, Type A intercalated cells and collecting duct principal cells are identified as pivotal in the transition from AKI to CKD. This process encompasses a variety of complex pathophysiological mechanisms, including inflammation, fibrosis, and renal tubular injury. Type A interstitial cells, a distinct group of cells located in the kidney’s collecting duct, are crucial for maintaining acid-base equilibrium and facilitating ion transport (76). AKI is frequently associated with an inflammatory response, which stimulates the release of pro-inflammatory cytokines and chemokines (77). Type A interstitial cells may exacerbate renal fibrosis by promoting fibroblast activation and collagen synthesis (78). Furthermore, the dysfunction of intercalated cells is intricately associated with alterations in the renal microenvironment, which may encompass hypoxia, modifications in the extracellular matrix, and dysregulation of intercellular signaling pathways (79). Collecting duct principal cells, another predominant cell type in the collecting duct, are responsible for the regulation of sodium and water reabsorption, thereby maintaining fluid balance (77). Dysfunction in the collecting duct principal cells results in compromised water and sodium reabsorption, further exacerbating kidney damage. Collecting duct principal cells demonstrate considerable proliferative capacity following acute kidney injury, a response likely aimed at compensating for tubular damage and facilitating renal repair (80). Consequently, these cellular types may represent potential therapeutic targets in the progression from AKI to CKD, warranting further in-depth investigation into the interactions among different cell types.

In this study, a biomarker-drug network was developed, revealing that CLCNKB, PLEKHA4, and KLK1 are concurrently targeted by tetrachlorodibenzodioxin. However, tetrachlorobiphenyldioxin is recognized as an environmental pollutant that induces toxicity across multiple tissues, including the kidneys (81). Research has demonstrated that exposure to tetrachlorobiphenyldioxin can result in oxidative stress, leading to cellular damage and dysfunction within the kidneys (82). However, the therapeutic effect of tetrachlorobiphenyldioxin are poorly studied. These findings are contrary to our results, indicating that the mechanisms of action of related drugs require further exploration in future studies.

In this study, miRNAs and immune cells synergistically influence the progression from AKI to CKD by targeting specific biomarkers and engaging in the inflammation-fibrosis axis. Regarding miRNAs, although only two miRNAs, such as hsa-miR-3187-3p, were predicted to target PLEKHA4, the constructed lncRNA-miRNA-mRNA network indicates its regulatory role. MiRNAs may negatively regulate PLEKHA4 expression by promoting mRNA degradation or inhibiting its translation. The downregulation or loss of function of miRNAs can lead to PLEKHA4 overexpression, which subsequently activates the Wnt/β-catenin pathway. This activation promotes fibroblast activation, epithelial-mesenchymal transition, and extracellular matrix deposition, thereby accelerating renal fibrosis (51). These findings illuminate the intricate mechanisms underlying immunometabolic regulation in kidney diseases and provide a rationale for therapeutic strategies targeting miRNAs or immune cells.

Among the biomarkers identified in this study, CLCNKB demonstrates significant novelty. Prior research has predominantly concentrated on the relationship between CLCNKB variants and inherited renal tubular disorders, such as Bartter syndrome (35). However, to date, no investigations have reported an association between CLCNKB and AKI or CKD. This study is the first to reveal that CLCNKB plays a crucial role in the transition from AKI to CKD, potentially offering a novel perspective on the mechanisms underlying AKI-CKD progression. In contrast, KLK1 and PLEKHA4 are established targets in AKI and CKD research. KLK1 has been demonstrated to play a significant role in kidney disease (83). Regarding PLEKHA4, the continuous activation of the Wnt/β-catenin signaling pathway is implicated in the progression of renal fibrosis in CKD, contributing to ongoing tissue damage in kidney disease (52). Through comprehensive bioinformatics analysis, this study systematically examined the expression patterns and potential regulatory networks of KLK1 and PLEKHA4 in the AKI-CKD transition, thereby enhancing the understanding of their mechanisms in kidney diseases. The identification of these biomarkers not only provides potential molecular indicators for early diagnosis but also enriches the current understanding of kidney disease pathophysiology.

Three biomarkers, CLCNKB, KLK1, and PLEKHA4, were identified through bioinformatics methods as being associated with the progression of AKI to CKD. Functional enrichment analysis was conducted based on these biomarkers to elucidate the biological pathways involved in AKI and CKD. Additionally, correlation analysis between differential immune cells and the identified biomarkers was performed to explore potential regulatory relationships. Single-cell analysis provided insights into the cellular-level expression of these biomarkers, offering new perspectives for early diagnosis and the development of novel therapeutic strategies for AKI and CKD. This study is subject to several limitations. Firstly, the retrospective analysis based on public databases is unable to fully eliminate batch effects, and the sample sizes are constrained (for instance, the scRNA-seq dataset includes only five cases of AKI and two cases of CKD), which impedes the effective application of multivariate statistical analysis to control for confounding factors. Secondly, the study lacks gene function experiments, such as gene knockout or overexpression, which are necessary to directly validate the causal mechanisms of the candidate genes. Furthermore, the clinical translation of target-related compounds, such as tetrachlorodibenzo-dioxins, is severely limited due to their toxicity. Future research should aim to expand the sample size through multi-center prospective cohort studies to acquire comprehensive clinical information. Additionally, animal models and cellular experiments, including gene editing and inhibitor or agonist treatments, should be employed to further elucidate the specific mechanisms by which CLCNKB, KLK1, and PLEKHA4 regulate fibrosis and the immune microenvironment. Moreover, flow cytometry and RNA sequencing (RNA-seq) technologies will be employed to assess the dynamic expression and functional status of type A interstitial cells within a kidney injury model, thereby elucidating their potential role in the disease pathology. Ultimately, these insights are intended to be translated into early intervention and targeted therapies for kidney disease through drug repositioning or the development of novel inhibitors or agonists.

## Data Availability

The datasets presented in this study can be found in online repositories. The names of the repository/repositories and accession number(s) can be found in the article/**Supplementary Material**.

## Supplementary material

Supplementary Figure 1 The quality control of AKI single-cell dataset. AKI: Acute kidney injury.

Supplementary Figure 2 The quality control of CKD single-cell dataset. CKD: Chronic kidney disease.
