# Supplementary figures and images for: Integrated transcriptomic and single-cell RNA-seq analysis identifies CLCNKB, KLK1 and PLEKHA4 as key gene of AKI-to-CKD progression
Source: Front Immunol. 2025 Sep 30;16:1628962. doi: 10.3389/fimmu.2025.1628962 (PMC12518305; doi:10.3389/fimmu.2025.1628962)

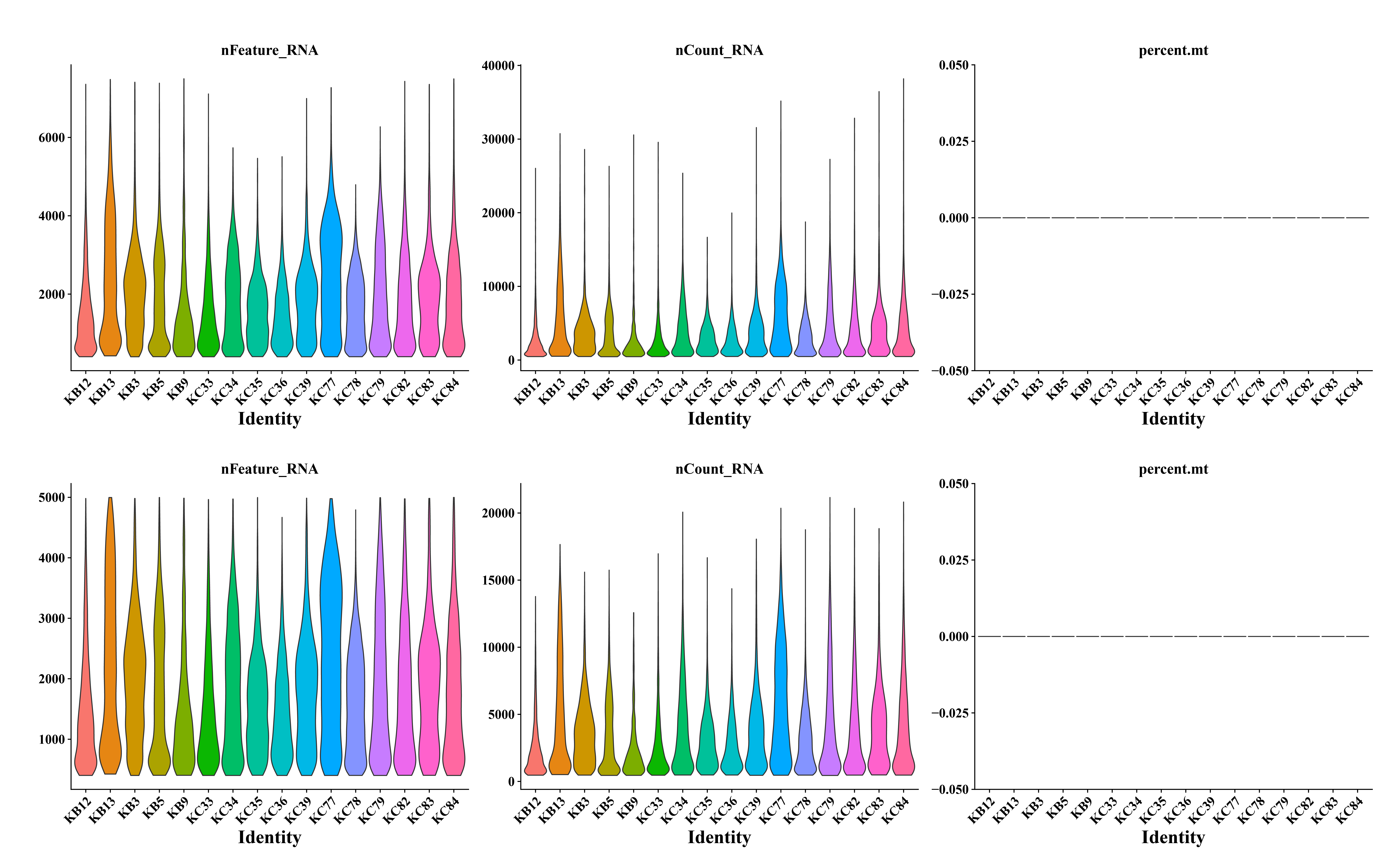

Supplement: Supplementary Figure 1 — The quality control of AKI single-cell dataset. AKI: Acute kidney injury. [file Image1.tif]

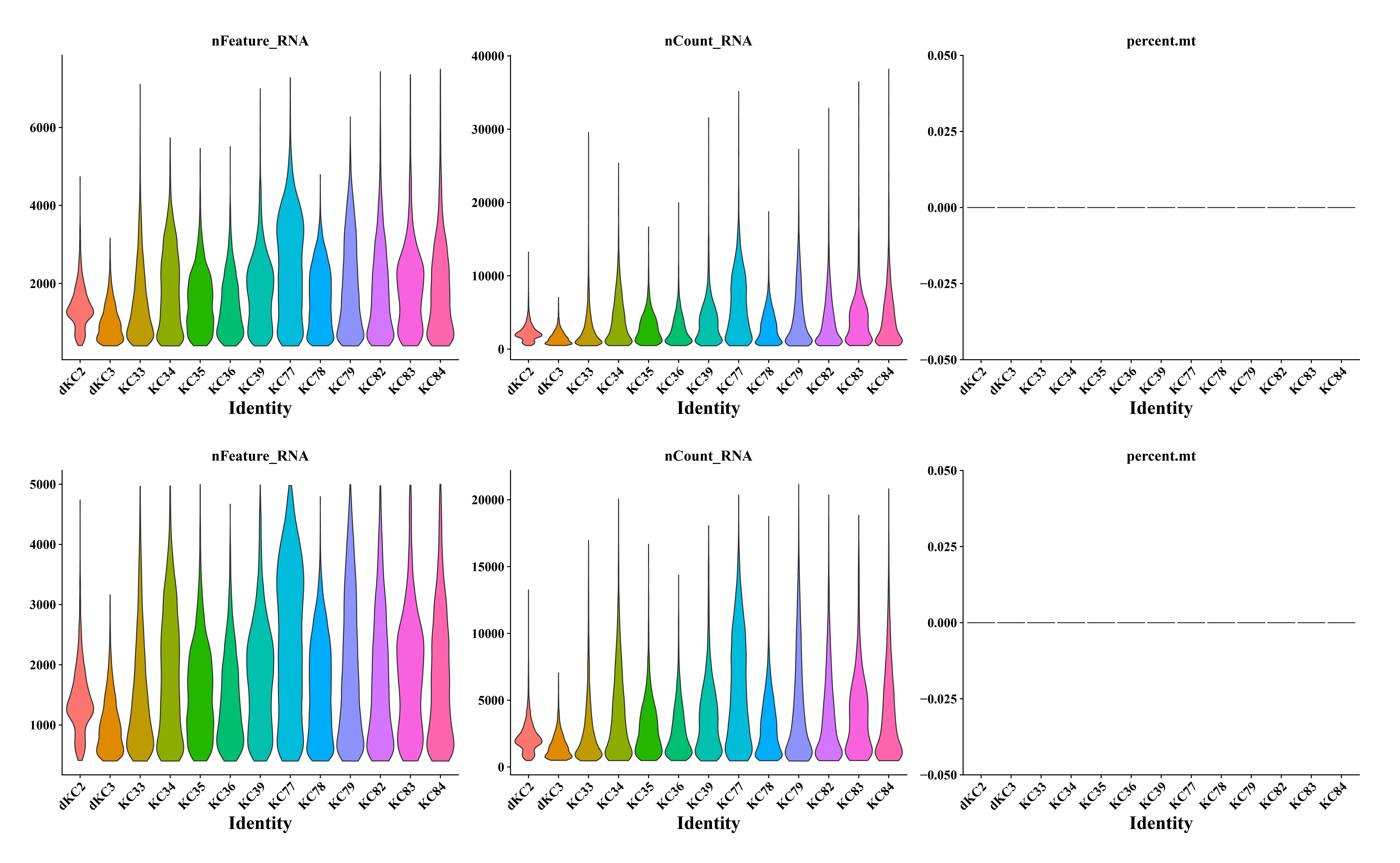

Supplement: Supplementary Figure 2 — The quality control of CKD single-cell dataset. CKD: Chronic kidney disease. [file Image2.tif]
